# *Pichia stipitis *xylose reductase helps detoxifying lignocellulosic hydrolysate by reducing 5-hydroxymethyl-furfural (HMF)

**DOI:** 10.1186/1754-6834-1-12

**Published:** 2008-06-11

**Authors:** João RM Almeida, Tobias Modig, Anja Röder, Gunnar Lidén, Marie-F Gorwa-Grauslund

**Affiliations:** 1 Department of Applied Microbiology, Lund University, P.O. Box 124, S-221 00 Lund, Sweden; 2 Department of Chemical Engineering Lund University, P.O. Box 124, S-221 00 Lund, Sweden; 3 Institut für Medizinische Mikrobiologie, Virologie und Hygiene, Universitätsklinikum Hamburg-Eppendorf, Campus Forschung, N27, 2. OG Martinistraße 52 20246, Hamburg, Germany

## Abstract

**Background:**

*Pichia stipitis *xylose reductase (Ps-XR) has been used to design *Saccharomyces cerevisiae *strains that are able to ferment xylose. One example is the industrial *S. cerevisiae *xylose-consuming strain TMB3400, which was constructed by expression of *P. stipitis *xylose reductase and xylitol dehydrogenase and overexpression of endogenous xylulose kinase in the industrial *S. cerevisiae *strain USM21.

**Results:**

In this study, we demonstrate that strain TMB3400 not only converts xylose, but also displays higher tolerance to lignocellulosic hydrolysate during anaerobic batch fermentation as well as 3 times higher *in vitro *HMF and furfural reduction activity than the control strain USM21. Using laboratory strains producing various levels of Ps-XR, we confirm that Ps-XR is able to reduce HMF both *in vitro *and *in vivo*. Ps-XR overexpression increases the *in vivo *HMF conversion rate by approximately 20%, thereby improving yeast tolerance towards HMF. Further purification of Ps-XR shows that HMF is a substrate inhibitor of the enzyme.

**Conclusion:**

We demonstrate for the first time that xylose reductase is also able to reduce the furaldehyde compounds that are present in undetoxified lignocellulosic hydrolysates. Possible implications of this newly characterized activity of Ps-XR on lignocellulosic hydrolysate fermentation are discussed.

## Background

Commercial production of bioethanol from lignocellulosic hydrolysate by yeast requires strains that (i) can ferment all sugars, both hexose and pentose sugars, in the hydroysate, and (ii) show sufficient tolerance to the inhibitors present in the hydrolysate [1,2]. Xylose-fermenting *Saccharomyces cerevisiae *strains have been constructed by heterologous overexpression of xylose isomerase (XI) or xylose reductase and xylitol dehydrogenase (XR/XDH) pathways (reviewed in [3]). While in the XI pathway, xylose is directly converted to xylulose, in the XR/XDH pathway xylose is initially reduced to xylitol by XR, and then xylitol is oxidized to xylulose by XDH. So far, efficient fermentation of xylose in lignocellulosic hydrolysates has been demonstrated for industrial *S. cerevisiae *strains carrying the XR/XDH pathway only [4-8].

In addition to hexoses and pentoses, the lignocellulosic hydrolysates may contain phenolic derivatives, acetic acid and the furaldehydes furfural and 5-hydroxymethyl-furfural (HMF) that inhibit yeast fermentation [2,9,10]. The effect on the metabolism in *S. cerevisiae *and the possible mechanisms conferring tolerance varies according to the nature of the inhibiting compound. For instance, tolerance to acetic acid is obtained by increasing ATPase activity, which pumps protons out of the cytoplasm [11], whereas tolerance towards furaldehydes and some phenolic derivatives is obtained by reduction of these compounds to less toxic alcohols [12-15]. Recently, a strong correlation between fermentation performances of *S. cerevisiae *strains in lignocellulosic hydrolysate and their ability to reduce HMF and furfural to furan-2,5-dimethanol (FDM) and 2-furanmethanol (FM) has been highlighted [13,16-18]. Up to now, two *S. cerevisiae *enzymes, the alcohol dehydrogenase 6 (ADH6) and an alcohol dehydrogenase 1 mutant (mut-ADH1) have been identified as enzymes responsible for the reduction of HMF and furfural in *S. cerevisiae *[12,19]. Overexpression of such enzymes in *S. cerevisiae *resulted in increased tolerance towards HMF and lignocellulosic hydrolysates [12,18,19].

The industrial xylose-fermenting strain TMB3400 was constructed by integration of *Pichia stipitis *XR/XDH pathway in the genome of USM21 followed by random mutagenesis [20]. In the current study, the fermentation performance of TMB3400 was compared with the parental strain USM21 in dilute acid spruce hydrolysate. The difference between the strains prompted us to investigate the putative role of *P. stipitis *XR (Ps-XR) in the response to furaldehyde inhibitors. *In vitro *and *in vivo *Ps-XR activity towards HMF was investigated in strains expressing Ps-XR at different levels and the kinetic properties of purified Ps-XR were also determined.

## Results

### Fermentation performance of TMB3400 and USM21 in spruce hydrolysate

**Figure 1:**
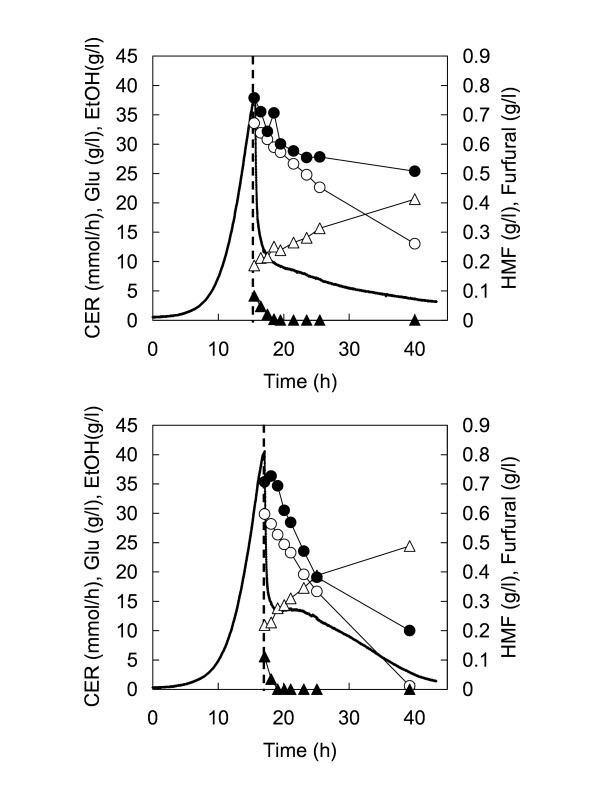
**Fermentation profile of the control strain USM21 (top) and the xylose-fermenting strain TMB3400 (down)  in batch fermentation of dilute-acid spruce hydrolysate**. Strains were grown in defined mineral medium until biomass concentration reached approximately 2.5 g/L. Then hydrolysate was added at approximately time 18 hours (dashed line). CER is represented by the continuous line. Furfural (▲), HMF (●), Glucose (○), Ethanol (△). Data for USM21 is taken from [16].

The fermentation performance of the industrial xylose-consuming strain TMB3400 was compared with its parental strain USM21 in dilute acid spruce hydrolysate. The strains were grown in defined mineral medium supplemented with glucose and once the biomass concentration reached approximately 2.5 g/L, a pulse addition of one volume of spruce hydrolysate (i. e. 300 ml) was added to the fermentor. After addition of the hydrolysate, carbon dioxide evolution rate (CER), glucose consumption, ethanol production and HMF and furfural conversion were followed (Fig. 1). Initially, the CER decreased rapidly for both strains. This is in agreement with what has been observed previously in similar types of addition experiments with spruce hydrolysates [16]. Thereafter, the CER was maintained at a constant level for approximately 8 h for strain TMB3400, whereas it continued to decrease for the control strain USM21 (Fig. 1). Clearly, strain TMB3400 had a higher fermentation rate in spruce hydrolysate, which cannot be explained by the fermentation of the low level of xylose that was present in the hydrolysate. During the first 8 h after hydrolysate addition, the average specific ethanol productivity for USM21 and TMB3400 was 0.21 (g/g/h) (data from [16]) and 0.35 (g/g/h), respectively. The higher ethanol productivity obtained with TMB3400 correlated with a higher HMF reduction rate, 0.016 g/g/h compared with 0.007 g/g/h for USM21 (data from [16]). The concentration of furfural was too low in the hydrolysate to determine any significant difference in furfural conversion.

### TMB3400 and USM21 reduction ability

**Figure 2:**
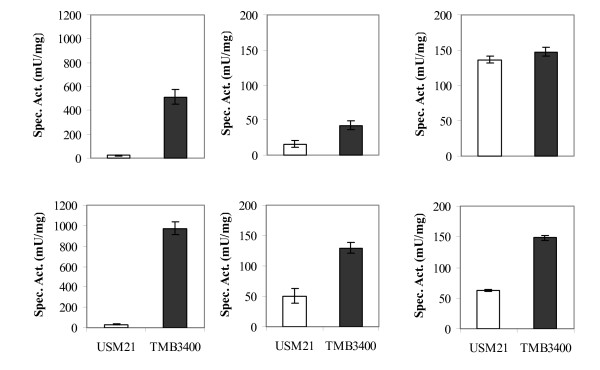
**Xylose (left graph), HMF (middle graph) and furfural (right graph) reduction activity measured in crude cell extracts from cells of strains USM21 and TMB3400**. Cells were grown aerobically overnight in defined mineral medium. The assays were performed using NADH (top) or NADPH (bottom) as cofactor. The values are averages of two independent measurements. The bars represent the deviation from the average.

The ability to ferment dilute acid hydrolysates has previously been associated with the ability to reduce furaldehydes [16-18]. For this reason, HMF and furfural reduction in strains TMB3400 and USM21 were compared by *in vitro *measurements using crude extracts from cells grown aerobically in glucose. In parallel, XR activity was measured as a control. As expected, TMB3400 showed high XR activity while USM21 only displayed background activity (Fig. 2). Furthermore, the cofactor preference of Ps-XR was confirmed, i.e. XR activity was higher when using NADPH rather than NADH as cofactor (Fig. 2) [21]. Both strains were able to reduce HMF and furfural; however TMB3400 showed higher specific activity in most cases (Fig. 2). TMB3400 was able to reduce HMF using NADH or NADPH as co-factor, whereas USM21 showed 3 times lower activity with HMF either using NADH or NADPH as co-factor. NADH-dependent furfural reduction was high in both strains, but TMB3400 showed approximately 3-fold higher NADPH-dependent activity with furfural than USM21 (Fig. 2).

### *In vitro* HMF and furfural reduction by Ps-XR

**Figure 3:**
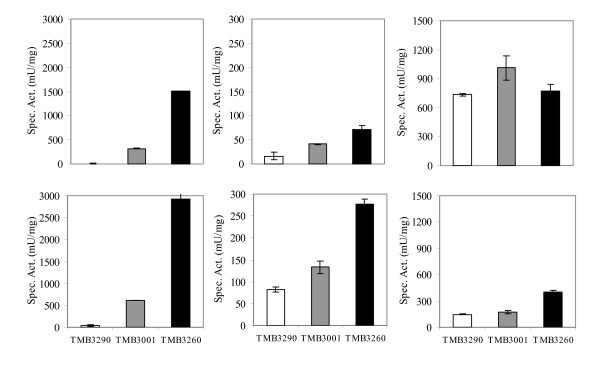
**Xylose (left graph), HMF (middle graph) and furfural (right graph) reduction activity measured in crude cell extracts from cells of strains TMB3290 (no Ps-XR), TMB3001 (low Ps-XR) and TMB3260 (high Ps-XR)**. Cells were grown aerobically overnight in defined mineral medium. The assays were performed using NADH (top) or NADPH (bottom) as cofactor. The values are averages of two independent measurements. The bars represent the deviation from the average.

Since the industrial strain TMB3400 was obtained from USM21 not only by targeted integration of the xylose pathway but also by further random mutagenesis, three *S. cerevisiae *strains derived from the same strain background without mutagenesis, which produce Ps-XR at different levels were used to confirm that Ps-XR was the enzyme responsible for HMF and furfural improved reduction in TMB3400. The control strain TMB3290 does not produce Ps-XR, whereas TMB3001 produces low level of Ps-XR and TMB3260 overproduces Ps-XR [22]. All three strains were grown aerobically in defined mineral medium supplemented with glucose and used to prepare crude cell extracts for enzymatic measurement of xylose, HMF and furfural reduction (Fig. 3). As expected, the control strain showed only background activity for xylose, whereas TMB3001 and TMB3260 shown increased specific activity against xylose using both NADH and NADPH (Fig. 3). Ps-XR was able to reduce HMF and furfural but the specific activity was approximately 6 times lower than with xylose (Fig. 3). The higher Ps-XR, the higher HMF and furfural activity. As for TMB3400 (Fig. 2), Ps-XR HMF reduction was coupled with NADH and NADPH whereas furfural reduction was coupled only with NADPH (Fig. 3). Like for xylose, NADPH-dependent HMF reduction activity was higher than NADH-dependent activity (Fig. 3). All strains presented a high NADH-dependent furfural reduction activity (~1000 mU/mg), independent of *Ps*-XR level (Fig. 3).

### *In vivo *HMF and furfural reduction by Ps-XR

**Figure 4:**
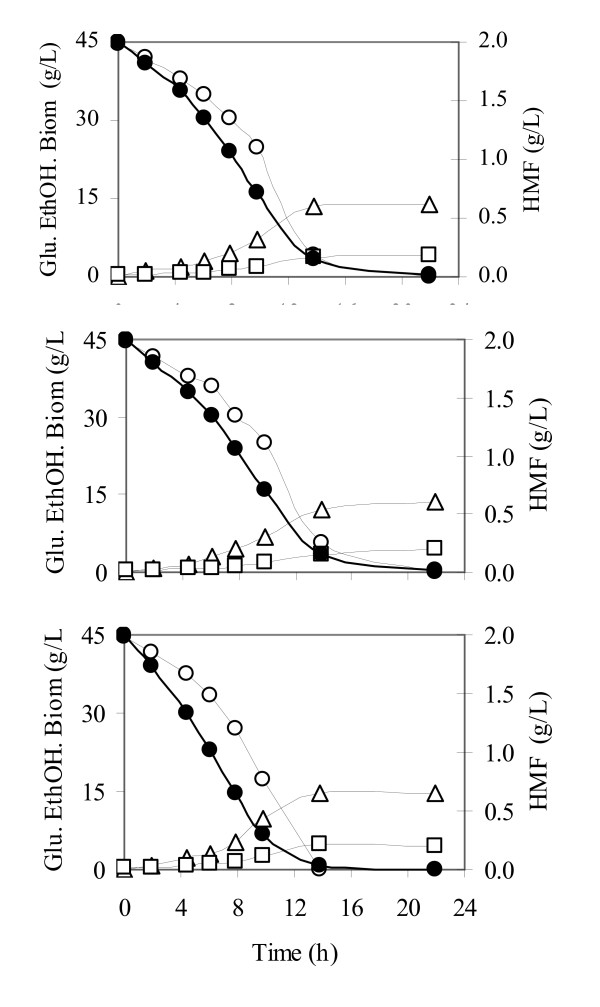
**Glucose batch fermentation with the *S. cerevisiae *strains TMB3290 (control – top), TMB3001 (low Ps-XR – middle) and TMB3260 (high Ps-XR – bottow) in the presence of 2 g/L HMF**. Glucose (○), HMF (●), biomass (□) and ethanol (△). The experiments were done in duplicate and the figure shows a representative profile for each strain.

In order to analyze the *in vivo *effects of Ps-XR overexpression in HMF and furfural reduction, batch fermentations in defined medium were performed with strains TMB3001 (control), TMB3260 (low Ps-XR) and TMB3290 (high Ps-XR) in the absence or presence of HMF or furfural. In the absence of inhibitor, all strains showed similar growth rates (Table 2) and product distribution (data not shown). In the presence of HMF, the control and TMB3001 strains presented similar grow rates whereas TMB3260 which overproduces Ps-XR had slightly higher growth rate and higher HMF reduction rate (Table 2, Fig. 4). In the presence of furfural, the growth rate of the three strains was also reduced but similar growth pattern and product distribution were observed for the three strains (data not shown).

**Table 1:** *Saccharomyces cerevisiae *strains used in the study.

**Strain**	**Description**	**Reference**
USM21	Polyploid industrial strain	[35]
TMB3400	USM21 (his3:: YipXR/XDH/XK) + random mutagenesis – produces XR, XDH, XK	[20]
CEN.PK 113-7A	Laboratory strain *MATa his3-Δ1 MAL2-8c SUC2*	[36]
TMB3290	CEN.PK 113-7A (*MATa his3-Δ1 MAL2-8c SUC2*) *his3*::YipXDH/XK – produces XDH, XK	This work
TMB3001	CEN.PK 113-7A (*MATa his3-Δ1 MAL2-8c SUC2*) *his3*::YipXR/XDH/XK – produces XR, XDH, XK	[34]
TMB3260	TMB3001 *PGK1p-XYL1 *– Overproduces XR	[22]

**Table 2:** Specific growth rate and HMF consumption rate for strains growing aerobically on glucose mineral medium supplemented or not with 2 g/L HMF.

	**No HMF**	**With HMF**	
		
	μ (h^-1^)	μ (h^-1^)	qHMF (g.g^-1^.h^-1^)*
Control	0.36 ± 0.01	0.20 ± 0.00	0.36 ± 0.02
Low Ps-XR	0.37 ± 0.00	0.20 ± 0.00	0.37 ± 0.02
High Ps-XR	0.36 ± 0.00	0.23 ± 0.01	0.45 ± 0.03

*Calculated after 6 hours.

### XR purification and kinetic characterization

Ps-XR was purified from the overproducing strain TMB3260 in order to determine the kinetic properties with HMF as substrate. The purification using gel filtration and affinity chromatography (Table 3) provided a 12-fold enrichment of the enzyme to a final specific XR activity of 8000 mU.mg protein^-1^. The purity of the enzyme was confirmed by SDS-Page. The kinetics of xylose and arabinose reduction by Ps-XR could be described by a Michaelis-Menten model (equation 1),

**Table 3:** Purification of *Pichia stipitis *XR from strain TMB3260 grown in defined mineral medium supplemented with glucose.

**Method**	**Amount Protein (mg)**	**Total Activity (U)**	**Specific Activity (U/mg)**	**Purification fold**
X-Press	725	478.5	0.66	1
Red-Sepharose	9	32.5	3.61	5.5
Gel Filtration	2.8	22.5	8.04	12.2

(1) $$\nu =\frac{\nu_{\max}s}{K_{m}+s}$$

in which the estimated Km values for xylose and arabinose 89.5 and 50 mM, respectively, were in agreement with previous reports [22,23]. In contrast, HMF reduction did not follow Michaelis-Menten kinetics as HMF concentrations above 60 mM appeared to strongly inhibit the enzyme (Fig. 5). Instead, the experimental data best fitted a substrate-inhibition model including a hill coefficient (Equation 2; Fig. 5) [24].

**Figure 5:**
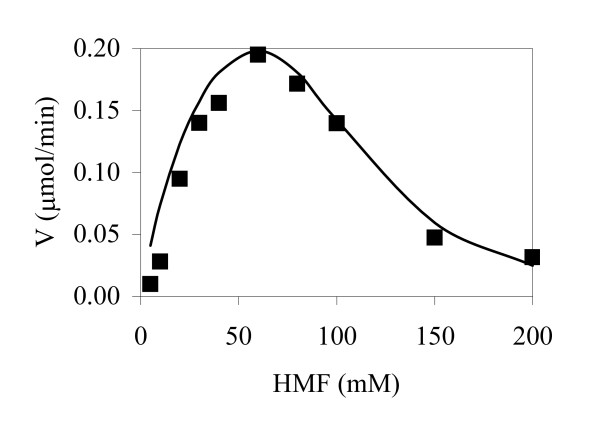
Measured (black boxes) and model (line) values of HMF conversion rate obtained with *Pichia stipitis *xylose reductase.

(2) $$\nu =\frac{\nu_{\max}}{\left(K_{m}/s\right)+1+{\left(s/K_{i}\right)}^{n}}$$

An inhibition coefficient (K_i_) equal to 95.0 and a hill coefficient (n) = 3.51 were obtained in the calculations for the model best fit The apparent K_m _and V_max _with HMF as substrate were 40 mM and 0.37 μmol min^-1 ^mg protein^-1^, respectively. The proportion of variance (R^2 ^= 0.95) given by the model indicated a good fit to the experimental data (Fig. 5).

## Discussion

*S. cerevisiae *NADPH-dependent alcohol dehydrogenase 6 (ADH6) and NADH-dependent alcohol dehydrogenase 1 mutant (mut-ADH1) have previously been identified as reductases able to reduce HMF, both *in vitro *and *in vivo *[12,19]. In this work, we demonstrate that *P. stipitis *xylose reductase (Ps-XR) can perform the same reaction using either NADH or NADPH. *P. stipitis *XYL1 gene encoding Ps-XR was the first XR gene to be efficiently expressed in *S. cerevisiae *[25-27] and Ps-XR was purified and the kinetic properties were determined for a wide range of substrates [21-23]. However, XR activity towards lignocellulosic inhibitors has never previously been reported.

As for ADH6 or mut-ADH1 [12,18,19], overexpression of Ps-XR in laboratory strains increased tolerance towards HMF and improved growth in the presence of the inhibitor. However, in contrast with ADH6 and mut-ADH1, Ps-XR could use both NADH and NADPH in HMF reduction, which may be favorable since strict use of NADH or NADPH by the alcohol dehydrogenases appear to change product distribution in defined mineral medium [18].

Despite higher *in vitro *HMF reduction activity for the laboratory strain TMB3001 than for the industrial strain TMB 3400, *in vivo *improvement of HMF conversion rate in laboratory strains was only evident with the highest Ps-XR activity (strain TMB3260). Variation in the HMF conversion rate and fermentation performance among the industrial and laboratory strains might be related with the experimental conditions. The industrial strains were evaluated under anaerobic conditions in hydrolysate containing media whereas the laboratory strains were tested under aerobic conditions in defined mineral medium supplemented with HMF. The industrial strains were exposed simultaneously to HMF, furfural, acetic acid and phenolic derivatives, which can have synergistic inhibitory effects on yeast [28]. Therefore the *in vivo *advantage given by Ps-XR may have been more easily distinguishable in TMB3400 than in the laboratory strains because the industrial strains were dealing and converting different compounds concurrently, with resulting low HMF reduction rate. The higher background for HMF and furfural reduction activities in the control strains might also have contributed to the absence of *in vivo *improvement of the laboratory strain with low Ps-XR level (TMB3001). The control strain USM21 had total furaldehyde reduction activity 3.5 times lower than the control TMB3290 (~270 mU/mg protein and ~940 mU/mg protein).

A possible role of Ps-XR in the *in vivo *conversion of other compounds like furfural and phenolics derivatives cannot be excluded. For instance, the *in vitro *Ps-XR ability to reduce furfuralwas demonstrated, but strains overexpressing this enzyme did not show any significant improvement in the fermentation performance in the presence of this inhibitor.

As demonstrated by kinetics studies, HMF inhibits alcohol dehydrogenase (ADH; EC 1.1.1.1), aldehyde dehydrogenase (AlDH; EC 1.2.1.5) and the pyruvate dehydrogenase (PDH) activities *in vitro *[29]. In our study, we demonstrated that HMF was also a substrate-inhibitor of Ps-XR, although the strongest inhibitory effects appeared when concentrations above 60 mM HMF were used. Considering that HMF concentrations in different hydrolysates vary between 10 mM and 40 mM, the HMF inhibition effects on Ps-XR are most probably not present *in vivo*. However, further studies may be required to analyze the *in vivo *Ps-XR response to HMF and the intracellular HMF level in yeast.

## Conclusion

We demonstrate for the first time that Ps-XR has furaldehyde reduction abilities, which helps *S. cerevisiae *detoxifying spruce hydrolysate. These results indicate a possible advantage in using XR instead of XI pathway for the construction of recombinant *S. cerevisiae *strains to be used in hydrolysates with high HMF content.

## Methods

### Yeast strains

*Saccharomyces cerevisiae *strains used in this work are listed in Table 1. The strains were maintained on agar plates containing yeast nitrogen base medium (YNB) (Difco YNB without amino acids 6.7 g/L) and 20 g/L glucose.

### Fermentation in spruce hydrolysate

The growth medium in inoculum cultures was a defined medium according to [30]. The hydrolysate used was produced from forest residue originating mainly from spruce in a two-stage dilute-acid hydrolysis process using sulphuric acid as the catalyst [31]. The composition of the hydrolysate was 24.3 g/l glucose, 12.1 g/l mannose, 2.9 g/l galactose, 5.6 g/l xylose, 1.4 g/l arabinose, 2.0 g/l acetic acid, 1.9 g/l HMF, and 0.5 g/l furfural. Adjustment of the hydrolysate to pH 5 with 6 M NaOH was made prior to use.

The inoculum cultures were grown in 300 ml cotton plugged shake-flasks containing 100 ml media supplemented with 15 g/l glucose in a rotary shaker at 160 rpm and at 30°C for 24 h. Batch fermentations were inoculated with 6 ml of the preculture and carried out in Belach BR 0.5 fermentors (Belach Bioteknik AB, Solna, Sweden). Initial concentrations of medium components were 2.67 times higher compared to the inoculum cultures in order to compensate for the dilution. The batch fermentations were started by growing cells on 30 g of glucose in an initial working volume of 300 mL. Next, a single addition of 300 ml of hydrolysate was made when growth reached mid/late exponential phase, which corresponded to a biomass concentration of approximately 2.5 g/l in the reactor. The stirrer speed and the temperature were 600 rpm and 30°C, respectively. The pH was kept constant at 5.0 by addition of 0.75 NaOH. Anaerobic conditions were obtained by continuously sparging the fermentor with 0.3 L/min nitrogen gas, which was controlled by a mass flow meter (Bronkhurst Hi-Tec, Ruurlo, The Netherlands).

### *In vivo *HMF and furfural reduction by Ps-XR

Inoculum cultures were grown overnight at 30°C in 250 mL cotton plugged shake-flasks with 25 mL of double-concentrated defined mineral medium (46) supplemented with 40 g L^-1 ^glucose and 200 ml L^-1 ^phthalate buffer (10.2 g/L medium KH phthalate, 2.2 g/l medium KOH). The same media was used in the growth curves, except for the addition of 2 g/l HMF where indicated. Growth curves were started at OD_620 _0.5 and were carried out in 100 mL media at 30°C in 1 L cotton plugged shake-flasks. The stirring rate was 200 rpm. Samples for biomass measurements were withdrawn regularly.

### Biomass and metabolites

Cell concentration was determined from absorbance measurements at 610 nm and dry-weight measurements were made from duplicate 10 ml samples, which were centrifuged, washed with distilled water and dried for 24 h at 105°C. The biomass concentration was correlated with OD by the dry weight measurements. The metabolite samples were immediately centrifuged, filtered through 0.2 μm filters and stored at -20°C until analysis. The concentrations of ethanol, glycerol and acetic acid were analyzed using HPLC system (Waters, Milford, Massachusetts, USA) equipped with Aminex HPX-87H column (Bio-Rad, Hercules, California) at 45°C. The mobile phase was 5 mM sulphuric acid with a flow of 0.6 ml/min. The concentrations of glucose, mannose, galactose, xylose, arabinose, HMF and furfural were measured on an Aminex HPX-87P column (Bio-Rad, USA) at 85°C, eluted with ultra-pure water at 0.6 ml/min. All compounds were detected with a refractive index detector, except for HMF and furfural which were detected with a UV-detector (210 nm). The carbon dioxide evolution rate was monitored on-line by measuring the concentrations of carbon dioxide and oxygen in the outgoing gas from the reactor with a CP460 gas analyser (Belach Bioteknik AB, Solna, Sweden). The gas analyzer was calibrated using a gas containing 20% oxygen and 5% carbon dioxide.

### Enzymatic activity measurements

Cell extracts were prepared with Y-PER reagent following the recommendations of the supplier (Pierce, Rockford, IL). The protein content in the cell free preparation was determined using Micro BCA Protein Assay Kit (Pierce). XR activity was measured in cell free extracts based on [32]. The reaction mixture contained 115 μM NAD(P)H and the reaction was started by adding 350 mM xylose in 100 mM Triethanolamine buffer (pH 7.0). Reductions of HMF and furfural reduction were measured as described in [33]. The reactions were performed in 100 mM phosphate buffer (pH 7.0) (50 mM KH_2_PO_4 _and 50 mM K_2_HPO_4_) and NAD(P)H was added to a concentration of 100 μM. The reaction was started by addition of 10 mM HMF or furfural. All assays were performed at 30°C and the oxidation of NAD(P)H was followed as the change in absorbance at 340 nm.

### Xylose reductase purification

Cells of TMB3260 from aerobic batch fermentation in 1.5 L defined medium [11] supplemented with 40 g glucose were used as starting material for the purification of XR. Once the culture reached early-stationary phase, the cells were harvested and used for Ps-XR purification following the protocol described in [22]. All purification steps were carried out at 4°C, and 5 mM 2-mercaptoethanol was added in all buffers to stabilize the enzyme activities. After each purification-step the enzyme activity of the different fractions were measured and the purification was proofed by SDS-gel-electrophoresis.

### Kinetic parameters

The kinetic constants for Ps-XR were determined using different substrates and NADPH as cofactor. The kinetic parameters V_max _(μmol min^-1 ^mg protein^-1^) and the Michaelis-Menten constant K_m _(mM) were estimated using non-liner regression analysis. The non-linear least-squares statistic tool from Microsoft-Excel (Microsoft) was used to parameter fitting. Typically, duplicate measurements at 10 different substrate concentrations spanning the K_m _value were used.

### Construction of TMB3290

Plasmid YIpXRXDHXK [34] containing the overexpression cassettes for XR and XDH from *P. stipitis *and xylulokinase from *S. cerevisiae *was cut with *Bam*HI. The restriction generated 3 fragments: 1.5 Kb XR cassette, 1.0 Kb *HIS3 *gene and the 12 Kb remaining plasmid. The *HIS3 *gene was re-ligated into the plasmid and used to transform DH5α *Escherichia coli *cells. The final plasmid YIpXDHXK, which does not express XR was used to transform *S. cerevisiae *CEN.PK113-7A. The positive clones were selected by growth in YNB media without amino acids and the strain was named TMB3290.

## Competing interests

The authors declare that they have no competing interests.

## Authors' contributions

JRMA performed the genetic work, enzymatic activity measurements, experiments with *in vivo *HMF reduction by Ps-XR, participated in the kinetic data analysis and drafted the manuscript. TM carried out the fermentation in hydrolysate and participated in the kinetic data analysis. AR performed the Ps-XR purification and participated in the kinetic data analysis. GL participated in the study design and its coordination. MFGG participated in the study design and its coordination and helped to draft the manuscript. All authors reviewed and approved the final manuscript.
